# Formulating and Deploying Strength Amplification Controllers for Lower-Body Walking Exoskeletons

**DOI:** 10.3389/frobt.2021.720231

**Published:** 2021-09-27

**Authors:** Gray C. Thomas, Orion Campbell, Nick Nichols, Nicolas Brissonneau, Binghan He, Joshua James, Nicholas Paine, Luis Sentis

**Affiliations:** ^1^ Department of Electrical Engineering and Computer Science, The University of Michigan, Ann Arbor, MI, United States; ^2^ Apptronik Inc., Austin, TX, United States; ^3^ The Human Centered Robotics Lab, The University of Texas at Austin, Austin, TX, United States

**Keywords:** exoskeletal assist system, force feedback, strength amplification, legged locomotion control, whole body control

## Abstract

Augmenting the physical strength of a human operator during unpredictable human-directed (volitional) movements is a relevant capability for several proposed exoskeleton applications, including mobility augmentation, manual material handling, and tool operation. Unlike controllers and augmentation systems designed for repetitive tasks (e.g., walking), we approach physical strength augmentation by a task-agnostic method of force amplification—using force/torque sensors at the human–machine interface to estimate the human task force, and then amplifying it with the exoskeleton. We deploy an amplification controller that is integrated into a complete whole-body control framework for controlling exoskeletons that includes human-led foot transitions, inequality constraints, and a computationally efficient prioritization. A powered lower-body exoskeleton is used to demonstrate behavior of the control framework in a lab environment. This exoskeleton can assist the operator in lifting an unknown backpack payload while remaining fully backdrivable.

## 1 Introduction

Exoskeletons offer the potential to greatly augment the physical load carrying ability by placing the strength of machines under the dexterous control of people. But the amplification of strength through force sensor feedback remains a challenging problem in practice. This problem is unique to this application area and is rarely discussed with regard to the various other types of exoskeletons—e.g., those that aim to recover locomotion capability lost to disease Kwa et al. (2009); Agrawal et al. (2017), offload the strenuous work of rehabilitation therapy from therapists Sugar et al. (2007); Kim and Deshpande (2017), or aid healthy locomotion with timed power boosts Mooney et al. (2014); Zhang et al. (2017); Sawicki et al. (2020). Amplification control systems are designed to magnify the physical strength of the operator as he or she interacts with a load through the exoskeleton, while also reducing the weight and inertia the operator feels from the exoskeleton itself. This kind of control allows non-repetitive, unpredictable tasks with unknown payloads.

Lifting known payloads is a simpler problem. These loads can be lifted by directly compensating their nominal weight with actuator torque commands (i.e., the “gravity compensation” strategy). This compensation could be lifting mostly the exoskeleton itself Kazerooni et al. (2005), or even offloading the operator’s own bodyweight Kong et al. (2010); Lv et al. (2018); Lin et al. (2019). In an exoskeleton system that can be easily backdriven by the operator, gravity compensation alone is a practical approach for lifting well-modeled payloads Campbell (2018). However, the operator must still accelerate the full inertia, compensate for any model error, and lift any extra payloads. The inertia burden can be lessened by adding positive acceleration feedback Kazerooni (2005); Kong and Tomizuka (2009), but all three issues can be addressed by adding force-feedback-based amplification.

Admittance control for exoskeletons Yu and Rosen (2013); Fontana et al. (2014); Jacobsen and Olivier (2014); Lecours et al. (2012) uses force sensor feedback at the human interface^1^ in order to increase the human-side closed-loop admittance, reduce sensitivity to the mass model, and lift unknown loads. But the admittance “increase” is relative to the admittance controller’s plant: a position-controlled robot. Since position-controlled robots have an artificially low admittance to begin with Yu and Rosen (2013); Gonzalez and Asada (2019), the closed-loop human-side admittance is typically not an improvement over the torque-controlled gravity compensation strategy. Additionally, the position-controlled plant of the admittance controller will attenuate all external forces acting on the robot. This has the disadvantage of depriving the operator of the force feedback they would normally perceive when they interact with the load.

In order to allow bidirectional transmission of forces to coexist with amplification of human strength, the exoskeleton must transmit both amplified forces from the user to the load and attenuated forces from the load to the user. And this requires a force sensor configuration that can distinguish between load- and human-originated forces. Directly measuring a robot–load interface and robot–human interface with force sensors allowed Kazerooni and Guo (1993); Kazerooni and Mahoney (1991a,b) to control disparate admittance behaviors for each interface.^2^ But the controller from Kazerooni and Guo (1993) was still not designed to improve the human-side admittance relative to the torque-controlled gravity compensation strategy. It still used admittance control and a position-controlled robot. In this paper, we use force sensing at the human–exoskeleton and actuator–exoskeleton interfaces (i.e., series elastic actuators), and this serves the dual purposes of distinguishing the human from the load and allowing torque control at the joints. The two interface admittances are then shaped with a cascade of amplification feedback on top of torque-controlled actuators.^3^

Unfortunately, the problem of non-passivity is inherent to feedback control that conceals inertia. This is an issue regardless of how the inertia was concealed—through positive acceleration feedback Kazerooni (2005) or force feedback Buerger and Hogan (2007). Without passivity, we must fall back to robust control in order to certify such behaviors. Most importantly, the exoskeleton’s human-facing port—its force–position relationship at the human–exoskeleton interface—will be in a feedback interconnection with the human’s exoskeleton-facing port. Studies of this feedback interconnection Kazerooni (1990); Buerger and Hogan (2007, 2006); He et al. (2019a) and the human in particular He et al. (2019b); He et al. (2020) have modeled the human as a mass-spring-damper system with a range of parameter values. The most variable parameter is stiffness, as this depends on muscle contraction Hogan (1984). We must demonstrate that no possible human behavior leads to instability—a robust control problem. Designing a machine to be passive Colgate and Hogan (1988); Hogan (1989); Colgate and Brown (1994); Adams and Hannaford (1999) can also be seen as a robust control problem: such designs guarantee stability against a very wide range of “human” behaviors—the set of all passive transfer functions. Our prior work Thomas et al. (2019); He et al. (2019a); He et al. (2019b); He et al. (2020) has studied this stability problem for a table-mounted elbow exoskeleton.

**FIGURE 1:**
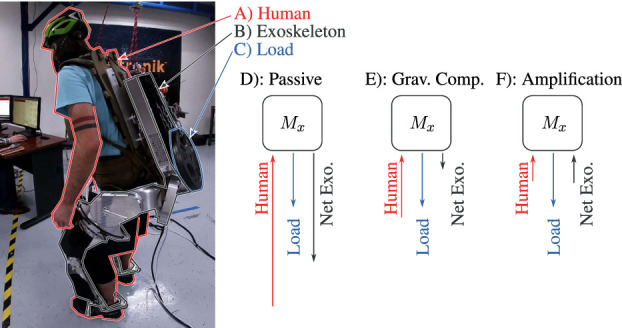
Human–exoskeleton–load interaction illustrating the concept of amplification. Marker **(A)**, the Human (inc. part of the backpack), connects to **(B)**, the Apptronik Sagittarius Exoskeleton, which connects to **(C)**, the unknown Load. The Human–Exoskeleton connection is force/torque sensitive to allow human force feedback. Three diagrams represent forces acting on the inertia matrix of the exoskeleton *M*_*x*_ in static equilibrium. When the exoskeleton is in zero-torque mode, the human supports both the load and the gravitational weight of the exoskeletons **(D)**. When the exoskeleton compensates gravity, the human needs only compensate the load and the gravity compensation error **(E)**. Amplification improves on this by making the exoskeleton augment the human input, in addition to compensating gravity **(F)**.

In this paper, we deploy an amplification controller on a 12 degree of freedom (12-DOF) lower-body exoskeleton with eight torque-controlled active joints and four passive (but sensorized) joints (Figure 1). The core framework of this controller is a multi-joint coordination approach modeled after humanoid robot controllers for torque-controlled joints Sentis et al. (2010); Kim et al. (2016) (e.g., the Valkyrie robot at NASA JSC Radford et al., 2015; Paine et al., 2015) in which a list of ‘tasks’ (e.g., the position of the end effector, or the force between the feet) is accomplished by the robot. The full controller comprises 1) an optimization that determines robot joint torques using a prioritized list of tasks and a set of constraints that act on the sum of human and robot torques—the “Shared-Body Controller” (Sec. 5); 2) a six degree of freedom (6-DOF) task that constrains the robot to follow human-led footstep transitions—the inter-foot force task (Sec. 4); 3) an amplification task that accomplishes strength amplification in Cartesian space (Sec. 2); and 4) a heuristic tuning strategy for the amplification filter, which is based on He et al. (2019a) and Thomas et al. (2019) (Sec. 3). We demonstrate the deployed controller’s ability to reduce the human effort necessary to lift the robot itself and an unknown payload, as well as the operator’s ability to easily back-drive the system to walk around and climb some stairs (Sec. 6).

## 2 Strength Amplification Task

Strength amplification can be illustrated using the example of an ideal fixed-base (arm-like) “exoskeleton” performing a force-feedback behavior with an end effector in contact with both the human and some load.

Consider a fully actuated, grounded base exoskeleton acted on by both a human operator (Jacobian *J*_*h*_, and force *f*_*h*_) and an unknown load (Jacobian *J*_*l*_, and force *f*_*l*_) (list of symbols in Table 1),

$$M_{x}\left(q\right)\ddot{q}+B_{x}\left(q,\;\dot{q}\right)+g_{x}\left(q\right)=\tau +J_{h}^{T}f_{h}+J_{l}^{T}f_{l}.$$ (1)

**TABLE 1:** Nomenclature for Sec. 2.

Symbol	Meaning
**C**, **R**, **Z**	Sets of the complex numbers, the real numbers, and the integers,
*n*_*q*_ ∈ **Z**	Dimension of the joint configuration space,
$M_{x}\in R^{n_{q}\times n_{q}}$ , $B_{x},g_{x}\in R^{n_{q}}$	Exoskeleton mass matrix, coriolis vector, and gravity vector
$\ddot{q},\dot{q},q\in R^{n_{q}}$	Exoskeleton joint acceleration, velocity, and position vectors
$\tau \in R^{n_{q}}$	(Fully actuated) joint torque vector
*n*_*h*_ ∈ **Z**	Dimension of the human interaction,
$J_{h}\in R^{n_{h}\times n_{q}}$ , $f_{h}\in R^{n_{h}}$	Human interaction cuff Jacobian, forces
*n*_*l*_ ∈ **Z**	Dimension of the load interaction,
$J_{l}\in R^{n_{l}\times n_{q}}$ , $f_{l}\in R^{n_{l}}$	Load interaction Jacobian, forces
*n*_*t*_ ∈ **Z**	Dimension of the task,
$J_{t}\in R^{n_{t}\times n_{q}}$ , $x_{t}\in R^{n_{t}}$	Task Jacobian, position
${\bar{J}}_{t}\in R^{n_{q}\times n_{t}}$	Dynamically consistent pseudo-inverse of *J*_*t*_
$\Lambda_{t}\in R^{n_{t}\times n_{t}}$	Task-space inertia matrix
${\hat{f}}_{t}\in R^{n_{t}}$	Desired task force
*α* ∈ **R**	Human strength amplification rate
${\hat{f}}_{a}\in R^{n_{t}}$	Ideal (infinite bandwidth) desired amplification force
*t* ∈ **R**, *s* ∈ **C**,	Time, Laplace variable
${\hat{f}}_{a}\left(t\right)\in R^{n_{t}}$ , ${\hat{F}}_{a}\left(s\right)\in C^{n_{t}}$	Desired amplification task force (time domain, frequency domain)
$f_{h}^{\prime }\left(t\right)\in R^{n_{t}}$ , $F_{h}^{\prime }\left(s\right)\in C^{n_{t}}$	Transformed human force (time domain, frequency domain)
$K\left(s\right)\in C^{n_{t}\times n_{t}}$	Force feedback filter

The exoskeleton measures the human forces, *f*_*h*_, and can use this measurement to specify *τ*. As we will see, by implementing an amplifying control law, the exoskeleton can reduce the human’s perception of the load. However to define this amplification law, we will need to first introduce the concept of a whole-body control task Sentis et al. (2010).

Whole body control tasks describe behaviors we want a robot to achieve, for example moving an end effector to a desired 6-DOF pose in Cartesian space. Tasks can also specify the desired internal forces of multi-contact Kim et al. (2016). More generally, tasks define both an effort-flow port of the robot and a target behavior for the robot to imitate at that port—a spring-damper behavior for position control and a force behavior for force control. This port is known as the task-space. By using the mapping between the joint-space of the robot and the task-space position *x*_*t*_ (and the mapping’s Jacobian, *J*_*t*_), a whole-body controller can implement the task behaviors even while floating in zero gravity or maintaining contact with arbitrarily shaped ground Sentis et al. (2010).

We define the amplification task to reduce human perception of load and exoskeleton dynamics disturbances in the task space. These task-space dynamics are originally (i.e., in open-loop) found by premultiplying (1) by ${\left(J_{t}M_{x}^{-1}J_{t}^{T}\right)}^{-1}J_{t}M_{x}^{-1}$ , yielding

$$\Lambda_{t}\left({\ddot{x}}_{t}-{\dot{J}}_{t}\dot{q}\right)+{\bar{J}}_{t}^{T}\left(B_{x}+g_{x}\right)={\bar{J}}_{t}^{T}\left(\tau +J_{h}^{T}f_{h}+J_{l}^{T}f_{l}\right),$$ (2)

where $\Lambda_{t}={\left(J_{t}M_{x}^{-1}J_{t}^{T}\right)}^{-1}$ is the task-space mass matrix and ${\bar{J}}_{t}=M_{x}^{-1}J_{t}^{T}\Lambda_{t}$ is the dynamically consistent pseudo-inverse of the task Jacobian Kim et al. (2016). The amplification task specifies only a linear subspace of the torque vector, ${\bar{J}}_{t}^{T}\tau$ , as

$${\bar{J}}_{t}^{T}\tau ={\hat{f}}_{a}+{\bar{J}}_{t}^{T}\left(g_{x}\right),\;\text{where}\;{\hat{f}}_{a}=\left(\alpha -1\right){\bar{J}}_{t}^{T}J_{h}^{T}f_{h}.$$ (3)

Here, the first term ${\hat{f}}_{a}$ represents a desired force amplifying the human operator’s strength, and the second term compensates gravity. Reduced human perception of load and exoskeleton dynamic disturbances can be seen in the closed-loop task-space,

$$\frac{1}{\alpha }\Lambda_{t}\left({\ddot{x}}_{t}-{\dot{J}}_{t}\dot{q}\right)+\frac{1}{\alpha }{\bar{J}}_{t}^{T}\left(B_{x}\right)={\bar{J}}_{t}^{T}J_{h}^{T}f_{h}+\frac{1}{\alpha }{\bar{J}}_{t}^{T}J_{l}^{T}f_{l}.$$ (4)

While the human term stays the same, every other term is reduced. Equivalently, we could say these closed-loop dynamics amplify the influence of the human force by a factor of *α*. But this behavior is complicated by the matrices ${\bar{J}}_{t}^{T}J_{h}^{T}$ and ${\bar{J}}_{t}^{T}J_{l}^{T}$ , which represent projection onto the space of the task as well as the potential for mismatch between the reference frames of the task, the human-measuring cuff interface, and the load.

In the special case where the human and load forces act only in the task-space and the human and load forces are expressed in the units and reference frame of the task-space (i.e., *J*_*t*_ = *J*_*h*_ = *J*_*l*_), this simplifies to

$$\Lambda_{t}\left({\ddot{x}}_{t}-{\dot{J}}_{t}\dot{q}\right)+{\bar{J}}_{t}^{T}\left(B_{x}\right)=\alpha f_{h}+f_{l},$$ (5)

which clearly shows the human advantage with respect to the load, inertia, and Coriolis forces. For example, this case occurs if 1) both forces are applied to one sensorized, 6-DOF end effector; 2) the sensor measurements of the spatial force vectors of the human and the load are all converted to the same reference frame Featherstone (2014); and 3) this frame is also the frame in which the task is expressed.

This law is unfortunately an unobtainable ideal, because it changes the apparent inertia the human feels instantaneously. In other words, the law requires that the actuation bandwidth is infinite. Beyond the actuation bandwidth, all feedback systems asymptotically revert to their natural dynamics. Thus, in the limit as frequency approaches infinity, the frequency-domain representation of exoskeleton torque should be zero.

### 2.1 Filtered Amplification Task

To allow for bandwidth limited amplification tasks, we introduce the notion of filtered force feedback amplification. Instead of an amplification task following Eq. 3, we define a desired filtered amplification force as the result of a frequency domain filter as

$${\hat{F}}_{a}\left(s\right)=K\left(s\right)F_{h}^{\prime }\left(s\right),\;\text{where}\;f_{h}^{\prime }\left(t\right)={\bar{J}}_{t}^{T}J_{h}^{T}f_{h},$$ (6)

$f_{h}^{\prime }$ is the human force vector represented in the task space, $F_{h}^{\prime }\left(s\right)$ is its Laplace transform, and *K*(*s*) is some matrix of linear filters analogous to (*α* − 1) in the ideal case. We design this matrix of filters to be diagonal, and consider a strategy for tuning the diagonal filter elements in Sec. 3.

In our exoskeleton, the amplification task is a 6-DOF task concerning the exoskeleton’s hip/backpack frame and the 6-axis force/torque sensor that connects the hip/backpack link to the operator. The vector *F*_*h*_′(*s*) is the 6-axis force/torque sensor measurement’s expression in the task frame (the exoskeleton’s hip frame). And ${\hat{F}}_{a}\left(s\right)$ is the desired amplification task spatial force vector (expressed in hip frame), which will be treated as a time-domain vector signal ${\hat{f}}_{a}\left(t\right)$ in Sec. 5.

## 3 Tuning the Amplification Filters

Since ideal amplification cannot be attained, we must consider a design space of more realistic amplification behaviors. And the essence of this design space is a bandwidth limitation on the control. This bandwidth limit, and its impact on coupled human–exoskeleton stability, has been studied in the context of single degree of freedom exoskeleton systems He et al. (2019a); Thomas et al. (2019); Huang et al. (2020); He et al. (2020), and we will use the single degree of freedom case as a heuristic for understanding the tuning of the amplification task’s **K**(*s*) filter elements in our multi-DOF exoskeleton. While this heuristic omits several obvious nonlinear effects and inter-task couplings in the full system, it captures the basic problem of human–exoskeleton instability that can occur when bandwidth limits are ignored.

### 3.1 Human-Exoskeleton Stability Model

**FIGURE 2:**
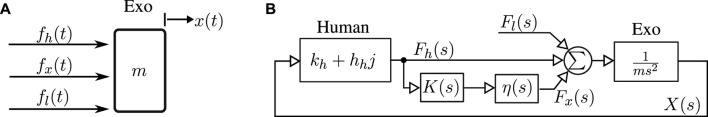
Amplification filter tuning model, a one-DOF mass (*m*) acted upon by human (*f*_*h*_), exoskeleton actuator (*f*_*x*_), and load (*f*_*l*_) forces **(A)**. Closed loop system resulting from complex stiffness human mechanical impedance and exoskeleton amplification with bandwidth-limiting time-delay and low pass filter effects in *η*(*s*) **(B)**.

Consider a 1-DOF linear human and exoskeleton system (Figure 2A; Table 2) where the exoskeleton acts like an inertia *M* and is being acted upon by three forces: the human *f*_*h*_(*t*), the actuator *f*_*x*_(*t*), and the load *f*_*l*_(*t*) as

$$m\ddot{x}\left(t\right)=f_{l}\left(t\right)+f_{h}\left(t\right)+f_{x}\left(t\right),$$ (7)

where *x*(*t*) the shared position of the human and the exoskeleton. We write this model in the frequency domain as

$$ms^{2}X\left(s\right)=F_{l}\left(s\right)+F_{h}\left(s\right)+F_{x}\left(s\right),$$ (8)

using capitalization to distinguish Laplace transforms from time-domain versions of the same signal.

**TABLE 2:** Nomenclature for Sec. 3.

Symbol	Meaning
*m* ∈ **R**	One-DOF exoskeleton mass
*k*_*h*_ + *h*_*h*_ *j* ∈ **C**	Human complex stiffness [see He et al. (2020)]
*x*(*t*) ∈ **R**, *X*(*s*) ∈ **C**	One-DOF position (time-domain, frequency-domain)
*f*_*l*_(*t*) ∈ **R**, *F*_*l*_(*s*) ∈ **C**	Load force
*f*_*x*_(*t*) ∈ **R**, *F*_*x*_(*s*) ∈ **C**	Exoskeleton actuator force
*f*_*h*_(*t*) ∈ **R**, *F*_*h*_(*s*) ∈ **C**	Human force
$\hat{\alpha }\left(s\right)\in C$	Desired amplification transfer function
*α*(*s*) ∈ **C**	Realized amplification transfer function
*K*(*s*) ∈ **C**	Feedback controller transfer function
*η*(*s*) ∈ **C**	Actuation imperfections (time-delay, low-pass filtering)
*ω*_*a*_ ∈ **R**	Amplification bandwidth (tuning parameter)
*α*_0_ ∈ **R**	Steady-state amplification rate
*ζ* ∈ **R**	Amplification damping ratio

The force-feedback filter *K*(*s*) is based on a nominal amplification behavior $\hat{\alpha }\left(s\right)=1+K\left(s\right)$ .^4^ We parameterize the desired amplification transfer function as

$$\hat{\alpha }\left(s\right)=\frac{s^{2}+2\zeta \omega_{z}s+\omega_{z}^{2}}{s^{2}+2\zeta \omega_{p}s+\omega_{p}^{2}},$$ (9)

i.e., a second order lag with two conjugate poles at lower frequency than the two conjugate zeros, using the same *ζ* twice for convenience, though this could potentially be optimized. While this $\hat{\alpha }\left(s\right)$ is not strictly causal, it produces a *K*(*s*) which is:

$$K\left(s\right)=\hat{\alpha }\left(s\right)-1=\frac{2\zeta \left(\omega_{z}-\omega_{p}\right)s+\omega_{z}^{2}-\omega_{p}^{2}}{s^{2}+2\zeta \omega_{p}s+\omega_{p}^{2}}.$$ (10)

Actuation imperfections ultimately limit the bandwidth, and we model these as the transfer function *η*(*s*). They include a time delay *T* and low pass filter effect (i.e., the closed loop bandwidth of the actuator’s torque controller) at frequency *ω* with damping ratio *ζ*,

$$\eta \left(s\right)=e^{-Ts}\frac{\omega^{2}}{s^{2}+2\zeta \omega +\omega^{2}}.$$ (11)

The mechanical impedance of the human is also modeled as a complex stiffness—a spring with a dissipation term that does not change with frequency. (This model can be interpreted as similar to a spring with a coulomb friction term that scales with the magnitude of deflection, such that the energy lost in flexing the spring does not depend on the speed of the flexing Brissonneau et al., 2021) This complex stiffness model is more accurate than the viscous damping model in predicting human energy dissipation in the elbow, especially at low frequencies He et al. (2020).

To facilitate easy tuning of our controller we reparameterize in terms of an amplification bandwidth parameter *ω*_*a*_ (equal to *ω*_*p*_) and a low frequency amplification gain *α*_0_ ≥ 1 (equal to $\omega_{z}^{2}/\omega_{p}^{2}$ ) so that

$$K\left(s\right)=\frac{2\zeta \left(\sqrt{\alpha_{0}}-1\right)\omega_{a}s+\left(\alpha_{0}-1\right)\omega_{a}^{2}}{s^{2}+2\zeta \omega_{a}s+\omega_{a}^{2}}.$$ (12)

This results in a closed loop system model (Figure 2B). We then tune only the amplification bandwidth *ω*_*a*_.

### 3.2 Tuning *ω*_*a*_

The tuning process we propose is simple. Sufficiently low values of *ω*_*a*_ are always stable, so we can increase *ω*_*a*_ until instability to tune the system without explicit system identification.

**FIGURE 3:**
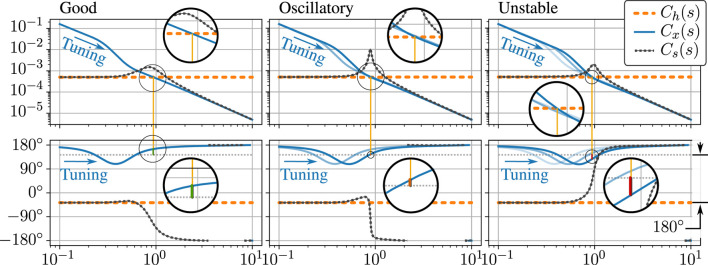
One parameter tuning of the amplification filter. Three bode plots show three different tuning configurations as the single tuning parameter (the amplification bandwidth *ω*_*a*_) is increased to failure in our frequency domain model. Tuning arrows indicate increasing *ω*_*a*_. Plotted are the (integral) admittance of the human, *C*_*h*_(*s*) = 1/(*K*_*h*_ + *C*_*h*_ *j*), the human-side admittance of the exoskeleton, *C*_*x*_(*s*) = 1/[*α*(*s*)*Ms* ^2^], and the admittance of the closed loop system obtained when the two are interconnected in parallel, *C*_*s*_(*s*) = *X*(*s*)/*F*_*l*_(*s*). Note that in the third plot, the phase of the closed loop system rises instead of falling, indicating an unstable pole in the right half of the complex plane. In all three bode plots, magnification is used to highlight the calculation of a “Human Phase Margin” which predicts this instability. This calculation uses the phase of *C*_*x*_(*s*) at the frequency where the magnitude of *C*_*x*_(*s*) is equal to the magnitude of *C*_*h*_(*s*)—the crossover frequency. At this frequency, stability of the resulting human–exoskeleton interconnection is determined by comparing the phase of *C*_*x*_(*s*) to a reference phase 180° offset from the phase of *C*_*h*_(*s*). The difference between the phase of *C*_*x*_(*s*) and this reference is the “Human Phase Margin.”

The bode plot of *X*(*s*)/*F*_*l*_(*s*) (“System” in Figure 3) transitions from a stable low-pass filter behavior to an unstable system as *ω*_*a*_ is increased. We note that the critical frequency satisfies a relationship akin to zero phase margin, where both the magnitude of the human (integral) admittance, 1/(*K*_*h*_ + *C*_*h*_ *j*) (“Human” in Figure 3), is equal to that of the amplified exoskeleton admittance, 1/[*α*(*s*)*Ms* ^2^] (“Robot” in Figure 3), and the phases of the two are offset by 180°. We call the phase angle difference *∠C*_*x*_ (*jω*) −*∠C*_*h*_(*jω*) − 180° at crossover frequency *ω* where |*C*_*x*_ (*jω*)| = |*C*_*h*_(*jω*)| the “Human Phase Margin.” Since the human phase margin is also the phase margin for the open loop transfer function $C_{x}\left(s\right)C_{h}^{-1}\left(s\right)$ (in the unit negative feedback case), the human phase margin predicts the stability of the closed loop system resulting from the human–exoskeleton interconnection.

A single tuning experiment can determine the limiting bandwidth for any particular amplification shape. Starting with *ω*_*a*_ very low, we slowly scale it up until the system appears to vibrate. Once the threshold of oscillation is found, the oscillation frequency is roughly the crossover frequency, and we could obtain an estimate of the human phase if we had a good model of the torque tracking performance and time delay. The problem is practically solved, however, by the formulation of the controller in a one-parameter tunable way. With one knob, it is easy to increase the performance up to the limit, back off for robustness, and get a good result in the end.

### 3.3 Practical Considerations

Ultimately this model is introduced as a heuristic for the tuning behavior occurring in the more complex exoskeleton system, so we now revisit its assumptions with an eye to the realistic case.

If a small value for *α*_0_ is selected such that the minimum phase of $1/\left(\hat{\alpha }\left(s\right)Ms^{2}\right)$ stays above the gray line in Figure 3, the system will be stable even for very high *ω*_*a*_. However, this will not hold true forever, and the bandwidth limiting factors in *η*(*s*) will cause the realized behavior 1/[*α*(*s*)*Ms* ^2^] itself to become unstable for high values of *ω*_*a*_.

The human model considered here neglected human inertia. If this term were added, the human inertia would be roughly comparable to the inertia of the exoskeleton. The model would not be changed at low frequencies, so the base case (stability of low *ω*_*a*_) of our tuning process would stay the same. The lower phase of the human due to the inertia would improve the maximum allowable *ω*_*a*_, but a limit would still exist due to the bandwidth limiting factors in *η*(*s*).

The inertia of the exoskeleton changes as the person moves it, and this means that the stability of the amplification behavior can change depending on the configuration. Practically, this means that when tuning for maximum performance, care will need to be taken to test each iteration of the *ω*_*a*_ parameter over a wide range of poses, to ensure a robust stability.

It is well known that humans have the ability to co-contract their antagonistic muscles and artificially raise their mechanical impedance, and this represents another changing aspect of this problem. If we assume that this scales both *k*_*h*_ and *h*_*h*_ together, as supported in He et al. (2020), then co-contraction will lower the human admittance and improve the human phase margin. In fact, by using a co-contraction predictor learned from wearable sensors (e.g., EMG and bicep circumference sensors), we have been able to design controllers that adapt online to take advantage of co-contraction to improve controller performance Huang et al. (2020). To ensure robust stability while tuning for performance, the operator will need to avoid co-contraction so as to explore the gain-limiting case.

## 4 Inter-Foot Force Task

Human-led foot contact transitions, such as walking or shifting balance, are an important part of any scheme for controlling lower-body exoskeletons. To allow this critical feature we introduce a second task, the inter-foot force task, that is achieved simultaneously and causes the exoskeleton to follow human-initiated foot lifting.

With one foot on the ground, this foot acts as a virtual base for the exoskeleton—a contact constraint on its otherwise free-fall dynamics. Since the exoskeleton is not designed to jump, we can assume that some sort of virtual base always exists. When two feet are on the ground at the same time, we can imagine a virtual single foot between them that acts like a base and moves between the feet according to the operator’s own weight distribution.

Two feet together have 12-DOF worth of reaction forces, and the virtual single foot contact only allows 6-DOF to be used as a virtual base. The remaining 6-DOF can be thought of as an error, representing the mismatch between the exoskeleton’s reaction force distribution and the operator’s. This error should be zero, and eliminating it is the purpose of the inter-foot force task.

To define this error, we first consider how correct reaction force distribution looks, and then consider the linear space of reaction forces perpendicular to this. For this purpose, we introduce an optimization problem that optimally distributes a net reaction wrench *f*_*s*_ into two components, one for each foot,

$$\begin{array}{ll} \underset{f_{1},\;f_{2}}{minimize}\; & \frac{f_{1}^{T}Q_{1}f_{1}}{2}+\frac{f_{2}^{T}Q_{2}f_{2}}{2} \end{array}$$ (13)

subjecttoX1*sf1+X2*sf2=fs, (14)

where *Q*_1_ and *Q*_2_ are positive definite and typically diagonal. We introduce two new reference frames: frame *s* (for “sum”), and frame *d* (for “difference”). Both frames are weighted averages of the two foot frames. Frame *s* is approximately matched with the human center of pressure. Frame *d* is the mirror image of frame *s*, and both frames overlap at the mid-foot point when the human puts equal weight on each foot. Transformation X1*s converts spatial force vectors from the 1st foot’s frame to frame *s*, and X2*s is the same for the other foot. The force *f*_*s*_ represents the sum of the two foot spatial force vectors expressed in frame *s* (Table 3).

**TABLE 3:** Nomenclature for Sec. 4.

Symbol	Meaning
*f*_*i*_ ∈ **R** ^6^	Foot *i*’s spatial force vector in frame *i* ∈ {1, 2}
*f* ∈ **R** ^12^	Stacking of *f*_1_ and *f*_2_
*f*_*s*_ ∈ **R** ^6^	Sum of foot spatial force vectors in frame *s*
*Q*_1_, *Q*_2_ ∈ **R** ^6×6^, *Q* ∈ **R** ^12×12^	Reaction force cost definition matrices
Xa*b∈R6×6	Spatial force vector transform, frame *a* → frame *b*
*λ* ∈ **R** ^6^	Lagrange multiplier vector in optimization
*X* ∈ **R** ^6×12^	Equality constraint matrix in optimization
Γ ∈ **R** ^6×6^	$={\left[XQ^{-1}X^{T}\right]}^{-1}$
$\bar{X}\in R^{12\times 6}$	= *Q* ^−1^ *X* ^*T*^Γ, a pseudo-inverse of *X*
*f*_*d*_ ∈ **R** ^6^	Inter-foot force task error in frame *d*
$\tilde{X}\in R^{12\times 6}$	Weighted inter-foot difference matrix
**G** ∈ **R** ^12×12^	Virtual base definition matrix

The equality constrained quadratic programming problem can be solved analytically. Starting from the equilibrium conditions,

$$\begin{array}{ll} & Qf+X^{T}\lambda =0,\;\text{where}, \end{array}$$ (15)

$$\begin{array}{ll} & Q=\left(\begin{array}{cc} Q_{1} & \\ & Q_{2} \end{array}\right), \end{array}$$ (16)

$$\begin{array}{ll} & f=\left(\begin{array}{c} f_{1}\\ f_{2} \end{array}\right), \end{array}$$ (17)

X=X1*sX2*s,and (18)

$$\begin{array}{ll} & Xf=f_{s}. \end{array}$$ (19)

In matrix form,

$$\begin{array}{ll} & \left(\begin{array}{cc} Q & X^{T}\\ X & 0 \end{array}\right)\left(\begin{array}{c} f\\ \lambda \end{array}\right)=\left(\begin{array}{c} 0\\ f_{s} \end{array}\right), \end{array}$$ (20)

$$\begin{array}{ll} & \left(\begin{array}{c} f\\ \lambda \end{array}\right)=\left(\begin{array}{cc} Q^{-1}-\bar{X}XQ^{-1} & \bar{X}\\ {\bar{X}}^{T} & -\Gamma \end{array}\right)\left(\begin{array}{c} 0\\ f_{s} \end{array}\right), \end{array}$$ (21)

where $\Gamma ={\left[XQ^{-1}X^{T}\right]}^{-1}$ and $\bar{X}=Q^{-1}X^{T}\Gamma$ . Thus

$$\begin{array}{ll} & f=\left(Q^{-1}X^{T}\right)\cdot {\left[XQ^{-1}X^{T}\right]}^{-1}f_{s}, \end{array}$$ (22)

f1f2=Q1−1X1*TsQ2−1X2*Ts⋅X1*sQ1−1X1*Ts+X2*sQ2−1X2*Ts−1fs. (23)

This 12-DOF solution *f* is the virtual single foot contact’s distribution of reaction forces between the two feet. The other six degrees of freedom in the foot forces—the degrees of freedom not specified by constraint Eq. 14—represent the inter-foot force task error. More specifically, we define the inter-foot force task error *f*_*d*_ in frame *d* to complete a parameterization of the foot forces *f*

$$f=\bar{X}f_{s}+\left[I-\bar{X}{\left({\bar{X}}^{T}\bar{X}\right)}^{-1}{\bar{X}}^{T}\right]{\tilde{X}}^{T}f_{d},$$ (24)

where we introduce

X~=X1*d−1w2−X2*d−1w1, (25)

as a rough parameterization of the deviation from the desired force distribution. This gets contorted into being perpendicular to $\bar{X}$ by the premultiplication with an $\bar{X}$ image space nullifier. As will be described in Sec. 5.5, the inter-foot force task puts a penalty on ‖*f*_*d*_‖, and when *f*_*d*_ = 0, reaction forces minimize the previously defined quadratic cost (since $f=\bar{X}f_{s}$ ). This leaves *f*_*s*_ as the path of least resistance the optimization uses to hold up the weight of the exoskeleton.

We define **G** to simplify notation:

$$f=G\cdot \left(\begin{array}{c} f_{s}\\ f_{d} \end{array}\right),$$ (26)

$$G=\left(\begin{array}{cc} \bar{X} & \left[I-\bar{X}{\left({\bar{X}}^{T}\bar{X}\right)}^{-1}{\bar{X}}^{T}\right]{\tilde{X}}^{T} \end{array}\right).$$ (27)

As mentioned in Sec. I, our exoskeleton controller is tasked with *simultaneously* accomplishing the amplification task at the hip/backpack interface (Sec. 3) and the inter-foot force task. In terms of reaction forces, the amplification task serves a similar purpose to the centroidal momentum task introduced in Koolen et al. (2016) or the center of mass task in Sentis et al. (2010): it determines the required sum of reaction forces. Meanwhile, the inter-foot force task [similar to the internal force tasks from Kim et al. (2016)] determines the part of the reaction force vector that is decoupled from the center of mass acceleration. With both tasks active, the reaction forces are all defined and the joint torques can be thought of as resulting from an inverse dynamics process—as in the Dynamic Balance Force Control of Stephens and Atkeson (2010).

## 5 Weighted 1-Norm Shared-Body Control

To combine the amplification task and inter-foot force task while also respecting limitations on the exoskeleton, we compute the joint torques of the exoskeleton using a linear optimization problem. This problem uses concepts of contact constraints, prioritization between task sub-components, a weighted 1-norm cost, and the actuator-mapped reaction force space in order to be computationally efficient.

### 5.1 Contact Constraints

Exoskeletons with feet can topple over, and we use an inequality-constrained floating base model Koolen et al. (2016); Kim et al. (2016, 2018); Mungai and Grizzle (2020) to keeping its feet flat on the ground. These inequality constraints act on the base–ground reaction forces, *f*_*r*_ defined by the combination of the floating-base exoskeleton and a contact constraint (notation as in Table 4):

$$\begin{array}{ccc} M_{x}\ddot{q}+B_{x}+g_{x}=S\tau +J_{h}^{T}f_{h}+J_{r}^{T}f_{r}+J_{l}^{T}f_{l}, & & \end{array}$$ (28)

$$\begin{array}{ccc} J_{r}\ddot{q}+{\dot{J}}_{r}\dot{q}=0, & & \end{array}$$ (29)

**TABLE 4:** Nomenclature for Sec. 5.

Symbol	Meaning
*n*_*q*_ ∈ **Z**	Dimension of the configuration space, inc. floating base (18 for sagit)
$M_{x}\in R^{n_{q}\times n_{q}}$ , $B_{x},g_{x}\in R^{n_{q}}$	Exoskeleton mass matrix, coriolis and gravity vectors
$\ddot{q},\dot{q},q\in R^{n_{q}}$	Joint acceleration, velocity, position
*n*_*τ*_ ∈ **Z**	Dimension of the torque space, sans floating base (12 for sagit)
$\tau \in R^{n_{\tau }}$	Optimization variable: joint torque vector
$S\in R^{n_{q}\times n_{\tau }}$	Underactuation matrix for a free floating base
$J_{h}\in R^{6\times n_{q}}$ , *f*_*h*_ ∈ **R** ^6^	Jacobian for human contact and forces
*n*_*r*_ ∈ **Z**	Dimension of the contact constraint (6 or 12)
$J_{r}\in R^{n_{r}\times n_{q}}$ , $f_{r}\in R^{n_{r}}$	Jacobian for ground contact and reaction forces
$C_{r}\in R^{20\times n_{r}},\;c_{r}\in R^{20}$	Reaction force inequality matrix and bias [see Thomas (2019)]
*e* (⋅) ∈ **R** ^12^	A task error function
*σ*(⋅) ∈ **R** ^12^	A task scalarization function
*s*_+_, *s*_−_∈ **R** ^12^	1-norm slack variables
*w* ∈ **R** ^12^	Weight vector
$J_{a}\in R^{6\times n_{q}}$ , $f_{a},{\ddot{x}}_{a}\in R^{6}$	Jacobian, force, accel. for the amplification task
$J_{f}\in R^{12\times n_{q}}$ , $f_{f},{\ddot{x}}_{f}\in R^{12}$	Jacobian, force, accel. for feet
$J\in R^{18\times n_{q}}$ , $f,\ddot{x}\in R^{18}$	Jacobian, force, accel. for composite task
$\bar{J}\in R^{n_{q}\times 18}$	Dynamically consistent pseudo-inverse of **J**
Λ ∈ **R** ^18 × 18^	Inertia matrix in composite task frame
**G** ∈ **R** ^12 × 12^	Virtual base definition matrix from Eq. 27
$\bar{\tau }\in R^{n_{\tau }}$	Maximum torque, human + exoskeleton
${\hat{f}}_{a}\in R^{6}$	Vector of filtered desired amplification task forces from Sec. 2

To avoid tilting the feet, sliding the feet, or pulling on the ground, we require

$$\begin{array}{ccc} C_{r}f_{r}+c_{r}\geq 0, & & \end{array}$$ (30)

where these inequalities include a simple approximations of the friction cone. For example, two rows would be used to express *μ*| *f*_*x*_| ≤ *f*_*z*_, where *μ* is the friction coefficient. The size of *C*_*r*_ depends on how many feet are on the ground. Joint torque limits are also represented as inequality constraints.

### 5.2 Actuator-Mapped Reaction Force Space

To speed up the solver and increase its accuracy, we reduce the number of free variables in our optimization problem by handling some equality constraints in advance. More specifically, we find ${\left(f_{a}{\left(\tau \right)}^{T},f_{f}{\left(\tau \right)}^{T}\right)}^{T}$ as functions of the optimization variable *τ*. Defining a new composite Jacobian, **J**, force vector, **f**, and task acceleration, $\ddot{x}$ , as

$$J=\left(\begin{array}{c} J_{a}\\ J_{f} \end{array}\right),\;f=\left(\begin{array}{c} f_{a}\\ f_{f} \end{array}\right),\;\ddot{x}=\left(\begin{array}{c} {\ddot{x}}_{a}\\ {\ddot{x}}_{f} \end{array}\right),$$ (31)

we can reformat the dynamics Eqs 28, 29 as a matrix equality with an analytic solution,

$$\begin{array}{ccc} \left(\begin{array}{cc} M_{x} & J^{T}\\ J & 0 \end{array}\right)\left(\begin{array}{c} \ddot{q}\\ -f \end{array}\right)=\left(\begin{array}{c} -B_{x}-g_{x}\\ \ddot{x}-\dot{J}\dot{q} \end{array}\right)+\left(\begin{array}{c} S\\ 0 \end{array}\right)\tau , & & \end{array}$$ (32)

which can be solved as in Eq. 21. We define the dynamically consistent pseudo inverse of **J** ^*T*^, ${\bar{J}}^{T}$ as

$${\bar{J}}^{T}={\left(JM_{x}^{-1}J^{T}\right)}^{-1}JM_{x}^{-1},$$ (33)

and the inertia in the composite task frame as $\Lambda ={\left(JM_{x}^{-1}J^{T}\right)}^{-1}$ . Together, we have

$$f=\Lambda \ddot{x}-\Lambda \dot{J}\dot{q}+{\bar{J}}^{T}\left(B_{x}+g_{x}\right)-{\bar{J}}^{T}S\tau .$$ (34)

Some terms in the previous expression are more significant than others, and some of the less significant terms are also corrupted by both imperfect knowledge of the exoskeleton’s mass matrix and (filtered) differentiation noise inherent in using quantized position sensors to estimate velocity and acceleration estimates. We did not notice a significant drawback in switching to a simplified version which represents a steady state equilibrium:

$$f={\bar{J}}^{T}\left(g_{x}-S\tau \right).$$ (35)

Of course, if we moved fast enough, these omissions would be noticeable. With this simplification, swinging the swing foot very fast should require the operator to resist the centrifugal extension of the knee due to the inertia of the exoskeleton. Also, squatting very quickly should result in a non-zero backpack sensor force due to the neglected acceleration terms. However, at the speeds we tested these effects were dwarfed by other control and mechanical imperfections. We hope that future exoskeletons will achieve such mastery over the basic terms that these dynamic terms will regain relevancy.

### 5.3 Prioritized Tasks

With multiple tasks and inequality constraints, the exoskeleton’s behavior is often over-specified. For example, the combination of the lateral (*y*-axis force) component of the amplification task, the mediolateral-plane rotation (*x*-axis torque) component of the amplification task, and the stance-foot’s lateral center-of-pressure limitation may require a non-zero task error. This is easy to visualize if the exoskeleton’s hip is far from the stance foot: the ground reaction force can point toward the hip and avoid rotation, or it can point straight up and avoid lateral force, but it cannot do both simultaneously. A more general version of this problem is well known in the humanoid robotics community Bretl and Lall (2008). This happens frequently during dynamic walking. And it demands that we specify not only which tasks we want to achieve, but in which order the task sub-components should fail to be satisfied if they conflict in this way.^5^

When constraints become active, there is neither an obvious choice for what to give up nor an analytical method to optimally decide. However, if we provide a prioritization of the task sub-components, then an optimal answer exists. This prioritization requires additional parameters—a rank order for each task sub-component—but these are relatively few, and easy to understand and adjust. This approach has also been used to handle redundancy in task definition even without the limitation of constraints Sentis et al. (2010). When constraints become active, the prioritization approach simply abandons the task sub-components one at a time, starting with the least important, until the problem is solvable. The lowest priority task sub-components are the ones for which we feel the human will have the easiest time comfortably handling the task sub-component failure. In the case of our amplification task, this could mean a failure to amplify the interaction force and/or a failure to achieve gravity compensation. In the case of our inter-foot force task, it could mean applying a force to the user’s swing foot (failure to gravity compensate) or failing to match the user’s desired contact force distribution (failure to transition appropriately, most evident if a foot is load-bearing when it should not be).

Strict prioritization between the tasks is a mathematically well-defined optimization scheme known as lexicographic optimization Bouyarmane and Kheddar (2017). Lexicographic problems must be solved as a series of related optimization sub-problems. First, the most important cost must be optimized within the problem constraints—the first optimization sub-problem. Next, the second most important cost must be optimized within both the original problem constraints and a new constraint. This new constraint requires that the previously minimized cost for the most important objective stays at its previously determined optimal value. With a solution to this second optimization sub-problem, a lexicographic optimization would proceed forward one cost at a time, solving optimization sub-problems with an ever-increasing list of constraints. And this recursive process will continue until each component of the prioritized list of costs has been optimized in its own sub-problem.

In our hardware, we could only solve three lexicographic optimization sub-problems within our 1 millisecond real-time control window, so with 12 prioritized task sub-components, a proper lexicographic solution was outside the realm of plausibility.

### 5.4 Weighted 1-Norm Cost

Weighted scalarization costs are an established approach to approximating a lexicographic optimization in the context of humanoid control Bouyarmane and Kheddar (2017). To avoid our computational bottleneck, we also used a scalarization that retains the linearity of the cost function. But in doing so we must add two positive slack variables and two inequality constraints for each scalarized cost (which were all task elements) to remain within a linear programming framework. For our vector of task errors *e*(*τ*), we define a vector of scalarizations *σ*(*τ*)

$$\sigma \left(\tau \right)=s_{+}+s_{-}\text{where}s_{+}\geq e\left(\tau \right),ands_{-}\geq -e\left(\tau \right),$$ (36)

where *s*_+_, and *s*_−_ are the newly introduced vector slack variables, and the new vector inequalities in Eq. 36 are elementwise inequalities. Under conditions that are almost always met,^6^ *σ*(*τ*) = |*e*(*τ*)| (as an elementwise absolute value).

This approach to modelling an absolute value function within the confines of a linear programming problem is the key to our application of a weighted 1-norm cost on the vector of all task errors. Clearly, summing the elements of *σ*(*τ*) results in the vector 1-norm of *e*(*τ*). Summing the elements of *σ*(*τ*) with positive weightings (setting cost equal to *w* ^*T*^ *σ*(*τ*) for some vector of positive weights *w*) is a weighted scalarization in the sense of Bouyarmane and Kheddar (2017), but we can also think of it as a weighted 1-norm—as the 1-norm for a scaled version of the original space. We prefer this as a name for the way it invokes a lozenge-like rhomboid geometry in 2D, and a diagonally-scaled octahedron geometry in 3D.

**FIGURE 4:**
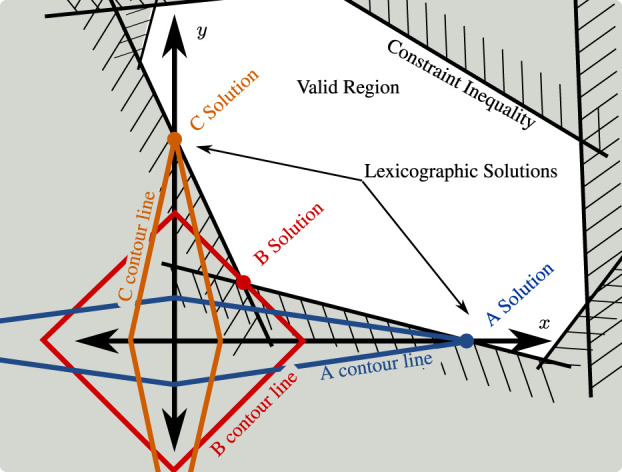
Illustration of how weighted 1-norm costs can behave similarly to lexicographic (prioritized) costs. Plot in the space of task error for task-*x* and task-*y*. Weighted 1-norm costs A, B, and C are depicted with a single contour line each. Optimal solutions for each task shown as colored circles. The so-called “sparsity promoting” nature of the weighted 1-norm cost can be understood in this context as optimal solutions which sacrifice one task to achieve the other. As exemplified by cost B, however, this is not guaranteed and depends on the inequality constraints and the shape of the valid region they generate.

To capitalize on this 2D intuition, Figure 4 illustrates how the weighted 1-norm cost can be adjusted through the weighting to approximate different lexicographic costs (there are only two in 2D space: either *x* matters more than *y* or vice versa). The illustration features a convex 2D set of solutions which satisfy constraints. The two axes represent orthogonal tasks, with the origin representing zero error for both tasks. Cost A uses a weighting that penalizes *y* error more than the *x*, cost B penalizes them roughly equally, and cost C penalizes *x* error more than *y*. In both cases A and C, the minimum cost point which satisfies constraints falls on one of the two axes—exactly as a lexicographic solution would. The fact that 1-norm costs tend to produce solution vectors with many zero entries (so-called “sparse” solutions Candes et al., 2008) is well known and frequently exploited. To promote lexicographic solutions instead of simply solutions with many zeros requires tuning the penalty weights to favor the prioritized tasks. In our illustration, the weightings in A and C are sufficiently extreme, and two lexicographic solutions emerge. Cost B illustrates a non-lexicographic middle-ground: neither cost is penalized enough to completely dominate the other, and the solution vector assigns non-zero error to both tasks.

One disadvantage of weighted 1-norm costs in exoskeleton control is that the constraints are continuously varying due to the changing exoskeleton geometry, and this can cause the optimal behavior to jump discontinuously. This can occur if the 1-norm cost discontinuously switches from being aligned with one lexicographic solution to a different lexicographic solution or even a non-lexicographic solution. We call these abrupt switches “priority inversion events.” To avoid these events entirely, we would need 1-norm weights with near-infinite scale differences between task sub-components. Since this is obviously not possible with floating-point numbers, the weighted 1-norm is an approximation: it sacrifices accuracy for speed. Fortunately, the approximation of the lexicographic problem is asymptotically perfect as the weight discrepancy increases. We exploited large differences in the weights to avoid priority inversion events during our experiment. The numerical precision of the linear program solver allowed us sufficient space to set these weights orders of magnitude apart and achieve reliable reproduction of the lexicographic problem in practice. These numerical limits restrict the total number of priority levels that can be correctly implemented.

### 5.5 A Linear Program for Shared-Body Control

At this point, we can express the optimization problem that the shared-body controller needs to solve at every controller update. Note that the passive joints^7^ are treated as being active joints for the purpose of the optimization. Their non-zero torques represent the expectation of the exoskeleton on the human operator.

We write our optimization problem,

$$\begin{array}{llll} & \underset{\tau ,s_{+},s_{-}}{minimize} & & w^{T}s_{+}+w^{T}s_{-} \end{array}$$ (37a)

$$\begin{array}{llll} & \text{ subject to } & & C_{r}f_{r}\left(\tau \right)+c_{r}\geq 0, \end{array}$$ (37b)

$$\begin{array}{llll} & & & \tau \leq \bar{\tau },\;-\tau \leq \bar{\tau }, \end{array}$$ (37c)

$$\begin{array}{llll} & & & s_{+}\geq e\left(\tau \right),\;s_{+}\geq 0, \end{array}$$ (37d)

$$\begin{array}{llll} & & & s_{-}\geq -e\left(\tau \right),\;s_{-}\geq 0, \end{array}$$ (37e)

with some new notation from Table 4. Slack variables *s*_+_ and *s*_−_ are introduced to describe absolute value operations. Weightings *w* form the weighted 1-norm cost. Limits on absolute torque are expressed with $\bar{\tau }$ . And the *τ*-dependent vector *f*(*τ*) from Eq. 38 (or from the steady-state approximation Eq. 39) is used to find *e*(*τ*) and *f*_*r*_(*τ*).

The first, *e*(*τ*), represents the 12-DOF vector of task errors for the amplification task and inter-foot force task:

$$e\left(\tau \right)=\left(\begin{array}{c} f_{d}\left(\tau \right)-0\\ f_{a}\left(\tau \right)-{\hat{f}}_{a} \end{array}\right),$$ (38)

where ${\hat{f}}_{a}$ is the desired amplification task force from (6) in Sec. 2.1; *f*_*a*_(*τ*) is the force the exoskeleton applies at the backpack interface, which is a part of *f*(*τ*) as written in Eq. 31; and *f*_*d*_(*τ*) is also related to *f*(*τ*) as in Eq. 26:

$$\left(\begin{array}{c} f_{s}\left(\tau \right)\\ f_{d}\left(\tau \right) \end{array}\right)=G^{-1}f_{f}\left(\tau \right),$$ (39)

using the matrix **G** from Eq. 27.

The second, *f*_*r*_(*τ*), represents the subset of the foot forces *f*_*f*_(*τ*) corresponding to the feet that are actually on the ground. This vector is used to compute the constraints associated with hard friction cones and unilateral contacts—i.e., Eq. 30, which is directly reproduced in Eq. 37b.

We call this program “Shared-Body Control” because the human and the exoskeleton’s torque and contact forces are both relevant. The true conditions for tipping over the foot are a function of both human and exoskeleton reaction forces. The sum of the human and exoskeleton reaction forces needs to lie within the friction cone, but sometimes the human works to counterbalance large torques the exoskeleton applies to the ground. We cannot know the human forces given our sensor configuration, so we are forced to be either optimistic (risking failure) or very conservative. Taking the conservative route means that our constraint will occasionally interfere with our tasks unnecessarily.

The human is also the only possible source of torques for the passive joints. By relaxing the torque requirements on the passive joints, the optimization will produce a torque vector representing a sum of exoskeleton and human originated torques. While we cannot expect the human to implement such torques, we can use this technique to prevent the exoskeleton from abandoning tasks which it could accomplish with help from the human (bounded, of course, by $\bar{\tau }$ ).

## 6 Implementation in Hardware

### 6.1 Hardware

**FIGURE 5:**
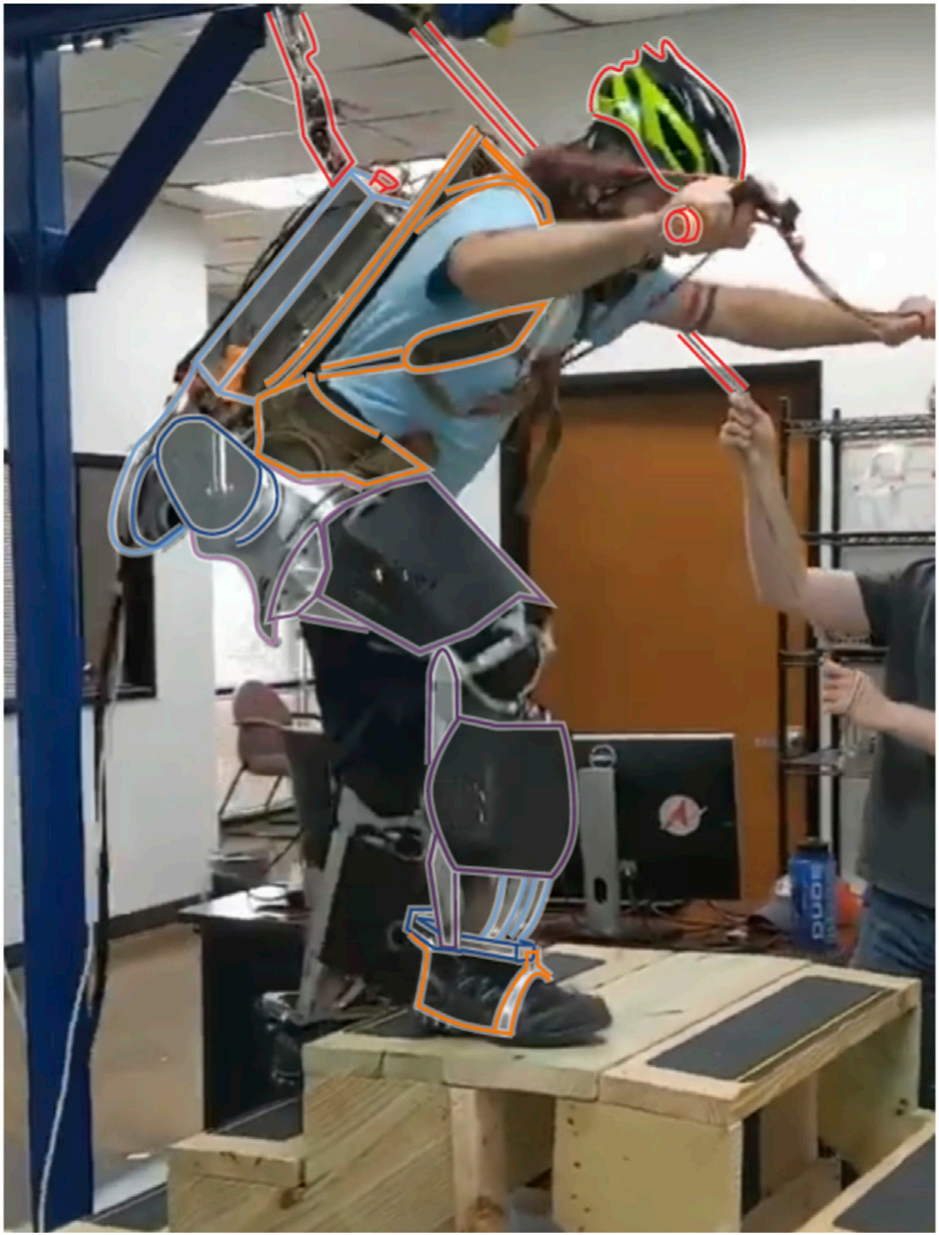
The Apptronik Sagittarius Exoskeleton used in this paper. The operator can climb stairs with the exoskeleton, even when it is not amplifying forces, due to the backdrivable torque-controlled actuators (gravity compensation and strength amplification are both active in the pictured movement). Coloring segregates rigid exoskeleton parts for the right leg (blue-through-purple), human interfaces (orange) and the safety features (red).

Our hardware platform is the Sagittarius P5 lower-body exoskeleton from Apptronik Systems, shown in Figure 5. This exoskeleton has 12 joints, six per leg. We name the joints in the serial kinematic chain from the torso to the foot 1) hip abduction/adduction, 2) hip flexion/extension, 3) hip internal/external rotation (hip yaw), 4) knee flexion/extension, 5) ankle flexion/extension, and 6) ankle pronation/supination (ankle roll). Of these six, four are powered joints. The two passive joints are hip internal/external rotation (also referred to as hip yaw for alignment with the local *z* axis) and ankle pronation/supination (which we also call ankle roll for similar reasons). The powered hip abduction and hip flexion joints are actuated by rotary series elastic actuators, while the other two feature proprietary linkage designs connecting linear series elastic actuators with rotary joint motion. Power is provided from off-board the device via a joint power and communication tether. The actuators communicate with a real-time Linux desktop workstation through an ethercat bus.

The different parts of the exoskeleton are highlighted in Figure 5, with rigid bodies being bordered by different color lines on the spectrum from blue to purple, human attachment points in orange, and safety features in red. To ensure the safety of the operator, the exoskeleton is attached via a slack safety rope to an overhead gantry system, and the rope’s height is operated by an assistant when the height is changing rapidly (as in the stair-climbing activities pictured in Figure 5. The operator wears a helmet, and there are multiple easy ways to stop the exoskeleton in an emergency: 1) a software emergency stop button, 2) a button on the top of the main backpack circuitry box, and 3) a button that the operator is required to hold at all times.

### 6.2 Controller Implementation

While we have presented the controller design in a very general way, not all of its nuanced behavior is relevant enough to demand implementation in the hardware system we used. In particular, the dynamic terms in Eq. 34 were not large enough for the operator to notice their omission, and the dynamically consistent pseudo-inversion of **J** is unnecessary given that **J** is invertible with the tasks we defined, thus

$$f=J^{-T}\left(g_{x}-S\tau \right).$$ (40)

Note that when a component of the amplification task has *K*(*s*) set to zero, it will not amplify human forces but will still compensate gravity.

We chose to amplify cuff forces at the hip/backpack sensor in order to assist the operator in lifting a backpack-mounted payload. And as previously indicated, we applied a inter-foot force task to allow the exoskeleton to switch ground contacts effectively. To summarize the tasks of the controller, the six individual spatial force vector components of the human-side force are fed into a diagonal matrix of amplification compensators as described in Sec. 3. And this occurs in the frame of the amplification task—the hip frame. For the three sagittal plane forces and torques (*x*-force, *z*-force, and *y*-torque) we may apply non-zero amplification, but the other three are left at zero in this work. This is based on the physical intuition that the sagittal plane forces and torque represent the larger interaction quantities during walking. This forms the 6-DOF amplification task. Based on a bed of 12 insole-mounted pressure sensitive resistors, a rough estimate of the human center of pressure is produced. This estimate is used to construct the elements of the inter-foot force task, which is also a 6-DOF task. With this hardware-specific preprocessing completed, the tasks are sent to a separate and more generic module to perform the linear programming optimization work. The software implementation of this optimization process is separate from the Apptronik control framework and is available as open source software Thomas (2019). It primarily acts as a wrapper layer for the linear programming solver from the COIN-OR Lougee-Heimer (2003) community.

### 6.3 Priorities

We avoided priority inversion events by iterative tuning of the priority weights (Table 5). This tuning was done with squatting and stepping behaviors similar to the planned tests. High-priority tasks that were never sacrificed held large weights, of roughly equal value. To effectively use the limited numerical precision, these tasks were equally ranked relative to each other. The most important weights were quickly identified and set to values that reliably avoided priority inversion in the tested behaviors. The more difficult question was identifying the priorities preferred by the operator.

**TABLE 5:** Implemented task priorities.

Task	Weighting
Hip amplification *x*-force	1 × 10^5^
Hip amplification *y*-force	1 × 10^5^
Hip amplification *z*-force	1 × 10^5^
Hip amplification *x*-torque	1 × 10^0^
Hip amplification *y*-torque	1 × 10^1^
Hip amplification *z*-torque	1 × 10^5^
Inter-foot *x*-force, limit penalty	1 × 10^–1^, 1 × 10^5^
Inter-foot *y*-force, limit penalty	1 × 10^–1^, 1 × 10^5^
Inter-foot *z*-force, limit penalty	1 × 10^–6^, 1 × 10^6^
Inter-foot *x*-torque, limit penalty	1 × 10^–6^, 1 × 10^5^
Inter-foot *y*-torque, limit penalty	1 × 10^–6^, 1 × 10^5^
Inter-foot *z*-torque, limit penalty	1 × 10^0^, 1 × 10^5^

We iterated various priority rankings between the components of the amplification task until our operator was satisfied with the behavior. First, we attempted to re-create linear inverted pendulum behavior by prioritizing the moment components over the force components. This prioritization had been effective with the Hume/Mercury biped robot Kim et al. (2016, 2020). Unfortunately, this first approach frustrated the operator, as the exoskeleton was naturally unstable. We eventually settled on the weightings in Table 5, which sacrifice *x*-torque first and are more comfortable for the operator. This preference may be exoskeleton or operator specific. The main drawback of the priorities from Table 5 is that at each stance transition the hips of the device roll such that the stance hip is higher than the swing hip—likely due to the lower penalty on hip amplification *x*-torque. However, we must sacrifice something, and this appeared to be the least-uncomfortable choice. The large swing in the hip position is due to the rather loose coupling that the backpack provides in this degree of freedom.

In testing, we began to suspect that operators may prefer a lower task penalty on the inter-foot force task while in double support but react strongly negatively to inter-foot force task violation while in swing (since this entails the exoskeleton loading their swing foot). We made a slight modification to the sum scalarized cost for the inter-foot force task as described in Eqs 37a, 37d, 37e, 38. A second copy of the task penalty was added, with a dead zone. We made the inter-foot force task error appear twice in the task error vector *e*(*τ*) instead of only once as in Eq. 38. Thus, we had two separate components of the weight vector *w* from Eq. 37a that penalized the same task. To introduce the dead zone for the second copy of the penalty, we added a sparse bias vector to 37d, 37e. We call this new penalty, with its dead zone and higher penalty cost, the “Limit Penalty” (see Table 5) since it acts like a soft limit forcing the values to stay within the dead zone. Within the dead zone, this new cost still behaves like the original weighted 1-norm cost (plus a constant bias that does not influence the optimum), but at the boundary of the dead zone, the weight suddenly becomes much higher.

We scheduled the dead zone width based on the center of pressure location, such that in single support this dead zone collapsed to zero and the inter-foot force task essentially took on the higher weighting of the limit penalty. In dual support, the width of the dead zone reached its widest when the feet were evenly balanced and reduced linearly in either direction away from that midpoint.

### 6.4 Demonstrating the Amplification Task

We conducted a set of simple tests to demonstrate the difference between gravity compensation and human strength amplification. These tests aimed to demonstrate an improvement in amplification stability relative to previous controllers developed for the exoskeleton and its previous partial prototypes (the 1-DOF testbed from He et al. (2019a); Thomas et al. (2019), a two degree of freedom leg, and a previous revision on the same lower-body design) under the same project Campbell (2018), which was a condition of our using the exoskeleton.^8^

**FIGURE 6:**
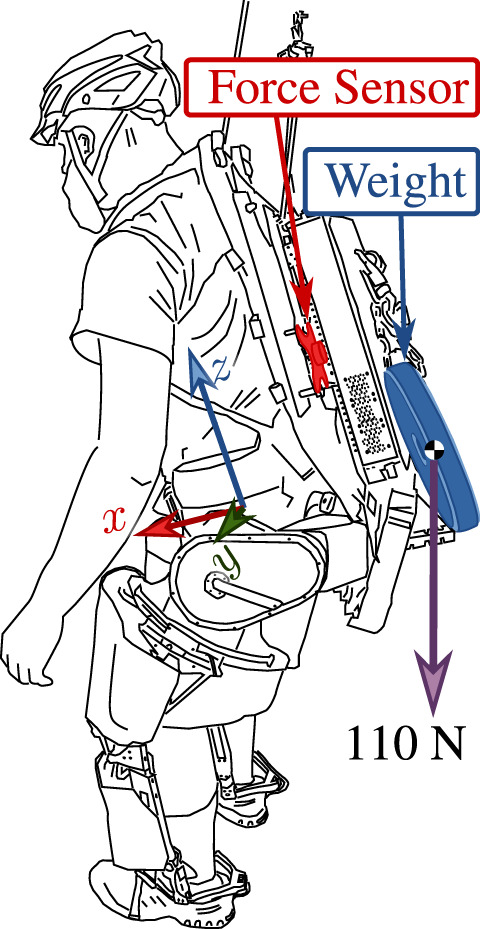
Load position in 6.4.3 and 6.4.4. Load hangs from a chain attached to the exoskeleton. Human effort measured with a six-axis force torque sensor, highlighted in red. Measurements are presented in the pictured “hip center” coordinate frame.

**FIGURE 7:**
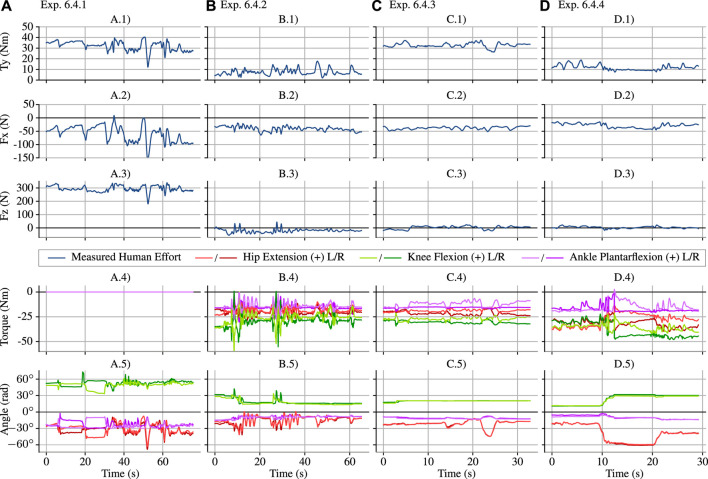
The four experiments from Table 6, shown as subfigure columns **(A**–**D)**, are compared in terms of the three sagittal plane components of the human–exoskeleton interaction force/torque, the sagittal joint torques, and the sagittal joint angles. In Exp. 6.4.1 **(A)**, the exoskeleton joints apply no torque (as shown in **A.4**), and the human–exoskeleton interface supports ≈300 N (as shown in **A.3**) as well as a ≈35 Nm moment at the hip (as shown in **A.1**). In Exp. 6.4.2 **(B)**, the controller is turned on with *α*_0_ = 1 (no amplification), and human–exoskeleton vertical force (**B.3**) and sagittal torque (**B.1**) are vastly decreased due to gravity compensation. In Exp. 6.4.3 **(C)**, a 11 kg mass is attached to the back of the exoskeleton (as shown in Figure 6), and this produces an increase in the human–exoskeleton sagittal torque (**C.1**), ≈30 Nm. Finally, Exp. 6.4.4 **(D)** increases *α*_0_ from 1 (no amplification) to 3 in the sagittal tasks, and the human–exoskeleton sagittal torque increase due to the added mass is reduced by roughly a third—considering **(B.1**,**C.1**,**D.1)** representing the average numerical value of the curves, D.1 − *B*.1 ≈ 1/3(*C*.1 − *B*.1)—as expected. With the amplification engaged, the operator deepens the squat at 10 s **(D.5)** and then moves to a second, less extreme squat at 20 s **(D.5)**, showing that the torque reduction continues to work. This squat is shown in the video attachment Thomas (2020). We would also expect that amplification would reduce the vertical force from the added mass; however, the vertical force remains roughly zero before adding the weight **(B.3)**, after adding the weight **(C.3)**, and with both the weight and amplification **(D.3)**—the expected 110 N force increase between **(B.3)** and **(C.3)** did not occur. Since the operator recalls feeling vertical forces from the addition of the mass, we suspect that there is a “force leak” where the vertical component transferred to the operator in a way the force sensor could not detect. Torque and angle measurements in the bottom two subfigure rows are measured using the exoskeleton’s spring deflection encoders and joint encoders, and therefore represent the exoskeleton’s—and not the operator’s—torque and position.

Figure 6; Table 6 show the basic structure of our tests: the operator wears the exoskeleton in a roughly standing position and various controller features are turned on and off. Extra weight is attached to the backpack as an unknown load in tests 6.4.3-4, and the image shows where it hangs relative to the operator. Figure 7 shows the results of the three tests. This experiment is shown in the video attachment Thomas (2020).

**TABLE 6:** Experimental parameters.

Test	SBC^a^	*α*_0_	Load (N)
6.4.1	Off	0	0
6.4.2	On	0	0
6.4.3	On	0	110
6.4.4	On	3	110

a —Shared-body controller (SBC) enabled.

In the first test, 6.4.1 the exoskeleton joints are on, but the desired torque is zero. The first column of plots in Figure 7 show the large *z*-force on the backpack due to the gravitational load of the exoskeleton acting on the operator. Variation in the angle shows that the operator was not perfectly holding still over the duration of the test. This natural human movement, while it prevents us from easily comparing across experiments (the operator does not even have the same resting posture between loading configurations), is hard to compensate for or avoid.

The next test, 6.4.2 enables gravity compensation—which means the torques from the shared-body controller are applied to the exoskeleton, but the amplification filters are all set to apply no strength amplification feedback (*α*_0_ = 1, so ${\hat{f}}_{a}=0$ ). This drastically reduces, but does not entirely eliminate, the interface forces and torques. Even if the exoskeleton’s mass parameters were perfectly modeled, the operator would still need to apply forces through this interface to control the passive joints of the exoskeleton. Compensating for the weight of the heavy exoskeleton is the most significant component of the system’s behavior. We can see this from the enormous reduction in human interface forces and torques in Figure 7 between 6.4.1 and 6.4.2: the vertical force, Fz, drops roughly 300 N, and the sagittal plane torque, Ty, drops roughly 40 N m.

In test 6.4.3, we added an 11 kg (25 lb) mass to the backpack, without changing the control mode. Based on our empirical determination, this represents the maximum load the exoskeleton could reliably handle without overheating during dynamic motions like walking. The test does not focus on the transient response but on the steady state behavior with the weight (mechanically, it would be hard to make the weight addition appear sudden without dropping it).

We see some unexpected behavior in the vertical sensor force: the weight’s 110 N did not transfer to the sensorized interface. The user confirmed that additional vertical force and sagittal torque were felt. This suggests a “force leak” in the design of the backpack sensor, where the force of the added weight is transferred to the operator without passing through the sensor. A likely culprit is the hip-pad of the backpack (directly connected to the operator) and the hips of the exoskeleton—as this would be consistent with the clear increase in the *y*-torque. The “force leak” does not appear to allow all vertical forces to bypass the sensor. 6.4.1 clearly shows large forces.

In the final test, 6.4.4, we engaged the amplification filters—providing a steady state amplification factor of 3, and a zero pair at 1 Hz for all three degrees of freedom in the sagittal plane. By choosing these conservative settings, we were able to achieve stability on the first try.^9^

Our system is pioneering in that it amplifies human strength at the backpack/hip link of the exoskeleton; there are no direct performance comparisons for this control feature. Our steady state amplification of human forces by 300% exceeded the 208% amplification (52% mass reduction) of sagittal hip moment in Zanotto et al. (2015), which also used force feedback to amplify human lower-body strength. However, this is not an exact comparison, as Zanotto et al. (2015)’s system used a treadmill mounted exoskeleton, had a different sensing configuration, and has only two degrees of freedom whereas our system has 12. The amplification’s pole frequency (0.58 Hz) and amplification magnitude (*α*_0_ = 3) at the hip/backpack human–exoskeleton interface are comparable to our previous results on a 1-DOF human elbow exoskeleton; in the notation of Appendix A, He et al. (2019a)’s robust controller used *α*_0_ = 10, *k*_*G*_ = 0.1, *Z*_*g*_ = 10, and *P*_*g*_ = 0.01, resulting in an amplification magnitude of 2.995 at 0.58 Hz. However, unlike our controller, He et al. (2019b) had even greater amplification at lower frequencies: its lowest pole-pair was at 0.146 Hz, and its steady state amplification rate was 9.91.

As shown in Figure 7’s fourth column, the human’s effort was reduced to roughly a third of its value in the third column in the *y*-torque component. More specifically, the disturbance due to the added weight, which can be seen by comparing 6.4.3 (with weight) against 6.4.2 (no weight) in terms of *y*-axis torque, is attenuated by the amplification factor, resulting in a much smaller disturbance effect when comparing 6.4.4 (attenuated weight) to 6.4.2 (no weight). We must make this comparison despite joint angle differences on the order of 10° between these tests—a limitation of our operator and operator–exoskeleton coupling. In 6.4.4, the operator engages in two different squat positions (switching posture at roughly 10 and 20 s). The interface forces remain within 10–15 Nm despite these kinematic changes. This supports the notion that if the operator were able to perfectly reproduce the posture from Exp. 6.4.3 in 6.4.4, the *y*-axis torque would also be within this range.

### 6.5 Demonstrating Foot Transitions

Distributing weight between the two feet using the inter-foot force task is a key behavior of the system and was tested when the operator walked on level ground and stairs. Since the exoskeleton itself was based on high bandwidth torque-controlled actuation, the operator could easily backdrive it to climb up stairs or to stand on one foot. While this happened, the exoskeleton continued to compensate for its own gravitational weight and amplify strength at the hip/backpack sensor.

**FIGURE 8:**
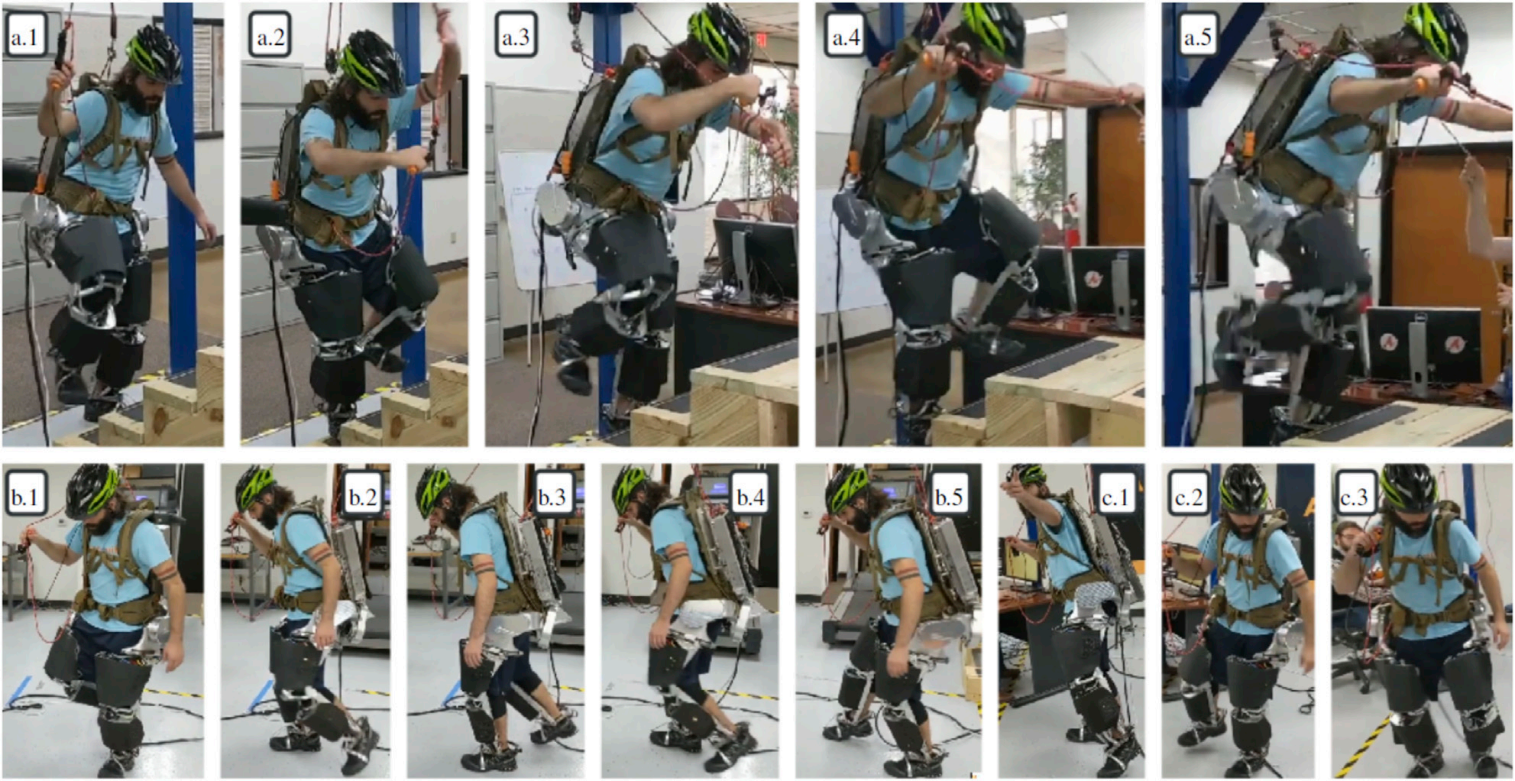
Frames from the demo. Frames **(A)**.1–5: climbing stairs with amplification but no added weight. Frames **(B)**.1–5: walking around. Frames **(C)**.1–3: walking around with amplification and extra weight.

**FIGURE 9:**
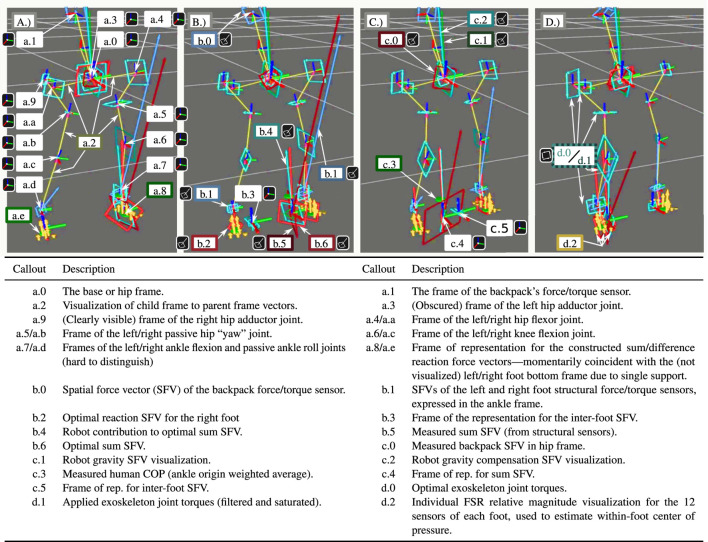
Human weight transfer in 0.2 s (subfigures evenly spaced in time) showing the exoskeleton visualization in the rviz program.

Figures 8, 9 show the operator shifting weight from one foot to another and lifting up the legs one at a time. Since the operator decreases the ground reaction force on a foot before lifting it, matching the measured human ground reaction force distribution between the feet leads the exoskeleton to reduce its own ground reaction force on that foot in anticipation of the loss of contact. As mentioned in Sec. 4, the weighting matrices *Q*_1_ and *Q*_2_ in Eq. 15 are scheduled according to the exoskeleton’s measurement of the human’s weight distribution. When the human shifts weight to one foot, the *Q* matrix penalty for reaction forces on the other foot becomes much larger. And since this causes the COP of the exoskeleton to approximate the COP of the human, this prevents the human from needing to lift a load-bearing exoskeleton leg. In addition, the penalty limit method allowed the exoskeleton more freedom during dual support but smoothly reduced this freedom when approaching single support, so that by the time it was reached the inter-foot force task was essentially the highest priority.

This behavior is shown in more detail through the internal exoskeleton visualization of Figure 9. This Rviz model visualizes many signals of interest, as described in the legend table. All frames are expressed as red (*x*) green (*y*) blue (*z*) line segments meeting at the local origin. Spatial force vectors (comprising a force and a torque) are shown as a ray from the local origin (the force) and a bi-vector—a directed plane comprised of four vectors making a square—to represent the torque. Joint torques are represented as pure bi-vectors. Unlike vector descriptions of torque, the bi-vector visualization has an unambiguous scaling relative to the force visualizations and cannot be confused for them. The four instants pictured in Figure 9 of the contact transition show the apparent center of pressure moving from the left foot to the right foot, and the corresponding shift in all the joint torques and the predicted reaction forces from the shared-body controller. As this is shifting, the reference frame of expression for the sum of reaction forces and the inter-foot force task’s difference of reaction forces swap feet. At all times, the reaction force/torque b.6 representing the sum is roughly equal to the sum ground reaction force calculated without using the passive joints b.4—which means that the exoskeleton is supporting the vast majority of its weight even during this transition. The backpack force/torque sensor b.0 confirms this, as it is small (and therefore hard to spot) throughout the transition.

## 7 Discussion

Strength amplification control offers us the potential to feel stronger as we manipulate the load through our exoskeleton. This paper deploys a control that has put that vision into practice under laboratory circumstances.

### 7.1 Benefits and Drawbacks

This controller has several advantages relative to the state of the art. It respects contact limitations—guaranteeing that the exoskeleton will never force the person to roll their ankles, lift their toes, or slide their feet. It improves human-side admittance relative to the gravity compensation baseline without the anti-stable acceleration feedback of Kazerooni et al. (2005). It keeps the human in control of the inter-foot force distribution using an elegant linear algebraic decomposition of the contact forces—a more general approach than Ref. Jacobsen and Olivier (2014). It allows the operator to move heavy objects without removing the force-feedback path that they would need in order to move the objects carefully—a force-feedback path that is removed by admittance control strategies Fontana et al. (2014).

Of course, the controller has downsides as well. The strategy depends on centralizing the contact between the human and the exoskeleton into a small set of sensors.^10^ This centralization places a significant burden on the mechanical design and introduces a new failure mode—the “force leak,” where interaction between the exoskeleton and the operator occurs outside the sensors. Additionally, all amplified interaction with the load must go through the exoskeleton structure—another mechanical design challenge. Due to the complexity of the mechanical design problem, the strategy makes it difficult to achieve the ultra-high energy density of successful locomotion augmentation exoskeletons Kim et al. (2019); Mooney et al. (2014). This is an open problem. Augmentation exoskeletons are already close to the energy-density boundary at which the energy they provide is equal to the energy they cost the user due to their mass. The extra design constraints make it harder for amplification exoskeletons to cross this boundary even at slow walking speeds.

### 7.2 Open Problems in the Control Framework

The control framework itself also has some open questions. Perhaps most pressing, is the lack of a formal stability guarantee that spans the entire control system from the human model to the inequality constraints of the linear program. In terms of the human model, we have approximated the mechanical impedance of the human and the cuff as being component-wise decoupled between the six degrees of freedom in our amplification task. Since an extremely low amplification bandwidth is still stable, and since our tuning process increases bandwidth until instability is discovered, this approximation limits us by introducing conservatism in the final tuning. Because of inter-component human coupling behavior, the tuning process may result in a different answer depending on the order with which the individual task sub-component bandwidths are tuned. In terms of the optimization, our approach may be at risk of chattering without a formal analysis of the hybrid-dynamical behavior of the discrete selection of an active set of inequality constraints and the continuous dynamics of the human and exoskeleton, though we have yet to observe this in practice.

Second, the framework was only tested with six amplification task sub-components. In theory, it supports arbitrarily many task sub-components. And it is also theoretically possible to join the inter-foot force task with the amplification task—to make the swing foot capable of acting like an amplified manipulator. Elimination of the inter-foot force *f*_*d*_ currently restricts the exoskeleton to applying a pair of ground reaction forces inside a six-dimensional space. The six-dimensional null space that is prohibited includes non-zero internal forces along the axis between the feet and canceling vertical torques perpendicular to the ground—the internal forces of multi-contact Sentis et al. (2010). If these internal forces were instead amplified, then the exoskeleton could theoretically assist in kicking and manipulation of objects with the feet. We lacked the sensing configuration for such a test: it would require the full 6-DOF interaction force/torque between the human foot and the exoskeleton foot to be measured, rather than just the vertical pressure between them. Thus, to validate the scalability our theory predicts, we would need an exoskeleton with either 1) more sensorized human contacts (arms, for example) or 2) the elimination of all human–load contact that does not pass through the exoskeleton as an intermediary.

Third, the controller tuning process is intended to be robust to all activities the operator performs, but we cannot know all these activities beforehand. A practical extension to this work would be to introduce an always-online learning process to continually adapt the tuning and avoid instability. Previously we have looked at tuning automation using online stiffness estimation Huang et al. (2020). However, this type of automation could potentially be simpler: if the system starts to vibrate, it could reduce the amplification bandwidth until the vibration subsides. Such a procedure would essentially automate our manual tuning approach.

On the other hand, higher performance might be obtained with a more complex strategy: modeling the human and redesigning the controller. Modeling the human online could exploit convex programs that automatically learn bounded-uncertainty models Thomas and Sentis (2019). With this more versatile system identification approach, even a human stiffness with “off-diagonal” terms could be learned. With every change to the model of the human stiffness bounds, robust control theory could synthesize a transfer matrix **K**(*s*) that guarantees stability.

Relating to the approximate lexicographic optimization using the 1-norm cost, other cost functions could also be considered. In particular, a 2-norm cost approach could smoothly transition through priority inversion events—improving over the hard-switching behavior of the 1-norm cost. Such a cost has been explored in Campbell (2018) for this exoskeleton and in Kim et al. (2020) for biped robots. However, such a cost did not realize a task priority, which hindered efforts to understand the required sacrifices when executing a behavior. Perhaps a generalizing compromise exists in costs that are locally quadratic, but asymptotically linear.

Finally, the approach makes an assumption that a foot is always on the ground—and this precludes interesting applications in free-fall, underwater (with neutral buoyancy), or micro-gravity. In such circumstances, the amplification task and inter-foot force task structures would need to be combined together and significantly altered. A “virtual single foot contact” would not exist. In its place, the change in centroidal momentum Koolen et al. (2016) would need to become the component of torque-space left intentionally unconstrained by the tasks. The remaining DOFs in torque-space would then be the subject of the new combined amplification task. The assignment of intuitive and easy-to-tune amplification controllers to such a task—which would concern an ever-changing subspace of the end effector contact force space—is an open problem. However, the approach to parameterizing the internal forces of multi-contact from Sentis et al. (2010) would be a reasonable starting point.

### 7.3 Series Elastic Actuators

Our exoskeleton hardware features series elastic actuators that are force/torque-controlled, and this decision also comes with benefits and drawbacks. To our knowledge, this paper is the first demonstration of Multi-DOF amplification control based on human interface force sensors and actuator force sensors (i.e., the series elastic elements). While such actuators are commonly used in wearable robots, they are a key part of our strategy, because with them we can avoid sensorizing the external force interface. This is a major advantage compared to systems designed to follow the extender concept Kazerooni and Guo (1993). The lack of load sensors gives us the freedom to properly handle amplification for load contract forces at any contact point along the structure of the exoskeleton.

As for series compliance itself, however, control performance would be better with nearly-rigid springs. In our experiment, the primary bandwidth-limiting factor that *η*(*s*) must describe is the 10 Hz bandwidth of the exoskeleton’s actuators. And this bandwidth is limited by the mechanical stiffness of the series spring, the noise level in the motor position and spring deflection sensors, and the bandwidth of the low-level electrical current controller. The time-delay of approximately 1 ms was non-limiting (due to the 10 Hz actuator bandwidth), so to improve the overall performance of the exoskeleton, the most efficient strategy would be to increase the spring stiffness and spring deflection sensor resolution. The series elastic actuators are simply torque sources to us, and direct drive motors offer higher bandwidth as torque sources. Removing the springs could also save weight. But series elasticity has some practical advantages: the force sensing is cheap and high quality, the exoskeleton’s motors are protected from impacts, and both the transmission’s friction and the rotor’s reflected inertia are well hidden from the user.

### 7.4 Potential Applications

We have demonstrated the control framework on the Apptronik Sagittarius exoskeleton, which is designed to lift heavy payloads as the user moves quickly. In this use case, the benefit of amplification control—relative to gravity compensation of the payload—is the potential reduction of inertial forces the user needs to compensate (without resorting to acceleration feedback) and the forces due to modeling error in the compensation. Future controllers for this application might investigate further enhancements to the operator’s quality of life, such as posture or safety support that guides the user.

However, amplification is also of great interest in load manipulation and heavy-duty tool use. We imagine some industrial amplification exoskeletons might be for slowly manipulating very large loads under direct human control. If they were to move fast, they would require significantly more impressive power density than we typically see today. Such an exoskeleton, worn by a skilled operator, might be fielded in difficult terrain as an alternative to tracked construction vehicles, perhaps with specialized tools for manipulating the environment. Given the strength of the system, these tools might not be constrained by weight relative to other tools for such difficult environments. The exoskeleton could act as an adjustable bracing system that allows the operator to maneuver them into position in a controlled way. For example, a construction worker could use an exoskeleton to maneuver an oversize pneumatic drill to carve a staircase on un-finished mountain terrain. Exoskeletons as platforms offer new possibilities for industrial tools and potential job sites by combining the flexibility of people with the strength of machines.

While our exoskeleton is designed to mimic the kinematics of the person wearing it, this is not the only way to approach the design. The control framework also has the potential to allow non-anthropomorphic exoskeletons to amplify human interaction. For example, consider a robot connected to an operator’s feet with long spindly legs that join together at a robot “hip.” Where this hip also features an enormous power tool that requires the user to manipulate it with both hands. Such an architecture would require the same control system features as our anthropomorphic exoskeleton structure: strength amplification in the frame of the robot’s hip, awareness of contact inequalities, and human-led footstep transitions.

## Data Availability

The raw data supporting the conclusions of this article will be made available by the authors, without undue reservation.

## Footnotes

1 Measuring human muscular effort, as can also be accomplished via electromyography (muscle measurement) Kawamoto and Sankai (2005); Young and Ferris (2016).

2 The HARDIMAN I exoskeleton Makinson et al. (1969) attempted to do this as well, but with a flawed approach that neglected multi-joint coordination.

3 Our reaction-force sensing series elastic actuators are torque controlled based on disturbance observers Paine et al. (2014); Paine (2014).

4 We reserve the symbol *α*(*s*) for the realized amplification behavior, which includes the effect of actuator imperfections *η*(*s*).

5 After trying both prioritizations, we determined that the operator prefers a failure in *x*-axis torque balance, even if this causes the exoskeleton’s hip to “wobble” relative to the human’s with every step due to the compliance of the backpack attachment in this degree of freedom.

6 Specifically that each element of *σ*(*τ*) appears in the cost with a strictly positive weight, and that *σ*(*τ*), *s*_−_, and *s*_+_ are otherwise decoupled from the problem.

7 The exoskeleton has two passive DOFs per leg: ankle inversion/eversion (ankle roll) and internal/external rotation of the hip (hip yaw).

8 Which is to say, our testing time was limited, and the scope of our experiments was narrow.

9 A later gain-tuning experiment revealed that the bandwidth limit is higher than this, but we ran out of time for exhaustive identification of this limit.

10 With one foot on the ground, our exoskeleton measures the human at two places: the hip/backpack attachment and the swing foot attachment.
